# Molecular Characterization of a Trisegmented Mycovirus from the Plant Pathogenic Fungus *Colletotrichum gloeosporioides*

**DOI:** 10.3390/v8100268

**Published:** 2016-09-27

**Authors:** Jie Zhong, Xi Dan Pang, Hong Jian Zhu, Bi Da Gao, Wen Kun Huang, Qian Zhou

**Affiliations:** 1 Hunan Provincial Key Laboratory for Biology and Control of Plant Diseases and Insect Pests, Hunan Agricultural University, Nongda road 1, Furong district, Changsha 410128, China; wzzhtx@sina.com (J.Z.); pangxd2016@163.com (X.D.P.); zjz0808@gmail.com (H.J.Z.); bdgao@aliyun.com (B.D.G.); 2 Institute of Plant Protection, Chinese Academy of Agricultural Sciences, Beijing 100000, China

**Keywords:** mycovirus, *Chrysoviridae*, *Colletotrichum gloeosporioides*, dsRNA virus

## Abstract

A novel double-stranded RNA (dsRNA) mycovirus, consisting of three dsRNA genome segments and possibly belonging to the family *Chrysoviridae*, was isolated from the filamentous phytopathogenic fungus *Colletotrichum gloeosporioides* and designated as Colletotrichum gloeosprioides chrysovirus 1 (CgCV1). The three dsRNAs of the CgCV1 genome with lengths of 3397, 2869, and 2630 bp (dsRNAs1–3) were found to contain a single open reading frame (ORF) putatively encoding the RNA-dependent RNA polymerase (RdRp), a capsid protein, and a protease, respectively, all of which exhibited some degree of sequence similarity to the comparable putative proteins encoded by the genus *Chrysovirus*. The 5′- and 3′-untranslated regions in each dsRNA segment contained similar sequences that were strictly conserved at the termini. Moreover, isometric virus-like particles (VLPs) with a diameter of approximately 40 nm were extracted from fungal mycelia. Phylogenetic analysis based on the conserved dsRNA1-encoded RdRp showed that CgCV1 is a new virus belonging to the *Chrysoviridae* family. BLAST analysis revealed the presence of CgCV1-like sequences in the chromosomes of *Medicago truncatula* and *Solanum tuberosum*. Moreover, some sequences in the transcriptome shotgun assembly (TSA) library and expressed sequence tag database (ESTdb) of other eudicot and monocot plants were also found to be related to CgCV1.

## 1. Introduction

Mycoviruses have been identified in almost all major groups of the kingdom Fungi, and an increasing number of novel mycoviruses have been reported [1,2,3]. The presence of many mycoviruses is associated with latent infections (appearing asymptomatic) in their hosts. However, some mycoviruses can incapacitate the virulence of their phytopathogenic fungal hosts, which may be exploited for biological control (e.g., the successful control of the chestnut blight in Europe) using the hypovirulent strains of its pathogen, caused by Cryphonectria hypovirus 1 (CHV1) [4]. The viruses in the family *Chrysoviridae* are commonly comprised of four monocistronic double-stranded RNA (dsRNA) segments ranging in size from 2.4 kbp to 3.6 kbp, separately encapsidated by non-enveloped isometric particles 35–40 nm in diameter. Based on representative members (Penicillium chrysogenum virus (PcV), and Helminthosporium victoriae 145S virus (HvV145S), the type species), viruses in the family *Chrysoviridae* often encode an RNA-dependent RNA polymerase (RdRp), capsid protein (CP), putative protease, and a protein of undetermined function [1,5,6]. Chrysoviruses, as well as other mycoviruses in the families of *Totiviridae* and *Partitiviridae*, infect not only fungi but also plants [7].

*Colletotrichum gloeosporioides* (Penz.) Penz. & Sacc., a plant pathogenic fungus, is commonly found on many angiosperm hosts. It is ubiquitous on citrus species and their relatives [8], causing young shoot and leaf blight, discolored leaf lesions, and stem-end wither, followed by premature fruit drop. Moreover, this fungus often produces decay from postharvest disease even after the fruit reaches maturity. Anthracnose occurs on dead and senescent tissues and especially manifests itself on the fruit surface, resulting in extensive postharvest losses [9]. Recently, *C. gloeosporioides* was also found to be the causal agent of postbloom fruit drop in Brazil [10] and Bermuda [11]. At present, any chemical approach to controlling this pathogenic fungus is complicated due to the long lifespan of *C. gloeosporioides* on citrus fruit, which may induce fungicide resistance and environmental pollution. Therefore, a biocontrol measure is a more feasible means of combating this fungal disease. With this aim, we investigated the presence of viruses infecting this fungus. To date, there are only a few examples of dsRNA elements or mycovirus infections in the *Collectotrichum* fungus [12]. Among these, isometric dsRNA-containing viral particles were isolated from *C. gloeosporioides* [13], and a novel gammapartitivirus has been identified in *Collectotrichum acutatum* [14]. In 2016, a mycovirus putatively belonging to the family *Partitiviridae* and a non-segmented dsRNA mycovirus showing close relatedness to the family *Partitiviridae* were also reported from the phytopathogenic *Colletotrichum* genus [15] and *Collectotrichum higginsianum* [16], respectively. In this study, we screened a large collection of *C. gloeosporioides* strains and discovered three dsRNAs of 2.5 kbp to 3.5 kbp in the HZ-1 strain isolated from an anthracnose-diseased citrus fruit. Molecular characterization indicated that these dsRNAs belong to a novel chrysovirus.

## 2. Materials and Methods

### 2.1. Fungal Isolate and Culture Conditions

*C. gloeosporioides* strain HZ-1 was collected from a diseased citrus fruit in Hanzhou, China. The isolate was maintained on potato dextrose agar (PDA; potato, glucose, agarose) at 27 °C. For dsRNA extraction, mycelial plugs were inoculated in potato dextrose (PD) broth (potato, glucose) in an orbital shaker (at 110 rpm) for 4 to 7 days at 27 °C.

### 2.2. Detection and Purification of dsRNA

Double-stranded RNAs were extracted using the methods described by Morris and Dodds with modifications [17]. The mycelial mass of strain HZ-1 was collected and ground in liquid nitrogen into a fine powder, with a mortar and pestle, and then transferred to an STE lysis buffer (100 mM NaCl solution, 50 mM Tris-HCl pH 8.0, and 50 mM ethylenediaminetetraacetic acid (EDTA; Sangon Biotech, Shanghai, China). The dsRNAs were extracted with phenol-chloroform (Solarbio, Bejing, China) and isolated with CF cellulose (Sigma-Aldrich, St. Louis, MO, USA) chromatography. The dsRNA nature of the extractions was verified by RNase-free DNaseI and S1 nuclease (TaKaRa, Dalian, China) treatments. The size was estimated by 2% agarose gel electrophoresis and visualized under an AlphaImager HP gel imaging system (ProteinSimple, Silicon Valley, USA). Each separated dsRNA segment was purified from the agarose gel using a MiniBEST Agarose Gel DNA Extraction Kit (TaKaRa, Dalian, China) and stored at −20 °C.

### 2.3. cDNA Cloning, Sequencing, and Phylogenetic Analysis

The complementary DNAs (cDNAs) of the purified dsRNA were obtained by reverse transcription and polymerase chain reaction (RT-PCR) amplification. A cDNA library was constructed using random hexadeoxynucleotide primers (TaKaRa). The gaps that were not covered by the cDNA library were filled by RT-PCR amplification, using sequence-specific primers designed from obtained sequences. To clone the dsRNA terminal sequences, a modification of a simplified single-primer amplification technique (SPAT) [18,19,20,21] was conducted. Briefly, the 3′-terminus of each strand of dsRNA was ligated to a 5′-end phosphorylated oligonucleotide Adaptor A (5′-PO_4_-TCTCTTCG TGGG CTCTTGCG-NH_2_-3′), using T4 RNA ligase (Fermentas, Vilnius, Lithuania) at 16 °C for 18 h. Then ligated dsRNA was purified and denatured for reverse transcription reaction with reverse transcriptase and Primer B (5’-CGCAAGAGCCCACGAAGAGA-3’) with sequences complementary to the oligonucleotide Adaptor A. The cDNAs of terminal fragments were then amplified using a Primer B and sequence-specific primers corresponding to the 5′- and 3′-terminal sequences of these dsRNAs. All of the amplified DNA fragments were cloned into a pMD-18 vector (TaKaRa, Dalian, China) and sequenced, with every base determined at least three times in each orientation.

Sequence similarity searches, using the BLAST program, were conducted in the NCBI database [22]. Sequence analysis, alignments, and phylogenetic analysis were carried out using the DNAMAN 6.0, CLUSTALX 1.8 [23], and MEGA 6 programs [24]. The bootstrap values for testing node robustness for the neighbor-joining (NJ) trees were calculated after 1000 re-samplings. Amino acid signatures and protein motifs were searched for with the Conserved Domains search included in the NCBI proteomics tools. RNA structures were predicted online using the DINAMelt Web Server (http://mfold.rna.albany.edu/?q=DINAMelt/Quickfold). BLAST (tBLASTn) searches, using each segment of Colletotrichum gloeosprioides chrysovirus 1 (CgCV1) to screen for virus-related sequences in eukaryotic genomes, were conducted against the NCBI databases (nucleotide collection (nr/nt)), genome survey sequences (GSS), high-throughput genomic sequences (HTGS), whole-genome shotgun (WGS) contigs, non-human, non-mouse expressed sequence tags (EST) transcriptome shotgun assembly (TSA), and others). Non-redundant hits, matching viral peptides with *E*-values <0.01, were extracted.

### 2.4. Preparation of Virus Particles

Purification of virus-like particles (VLPs) was performed. About 20 g of mycelium was ground in liquid nitrogen into a fine powder and then mixed with 120 mL of 0.1 M sodium phosphate buffer (pH 7.0). The suspension was homogenized and clarified in the presence of 10% chloroform (v/v) and *n*-butanol (1:1) by centrifugation (at 7000× *g*) for 30 min at 4 °C. The aqueous supernatant was adjusted to 8% polyethylene glycol (PEG 6000; Sangon Biotech, Shanghai, China) (w/v) and 0.1 M NaCl (w/v) and stirred on ice for 4 h. The precipitate was re-suspended in 0.01 M phosphate buffer (pH 7.0) after centrifugation (at 10,000× *g*) for 30 min. The suspension was re-centrifuged (at 10,000× *g*) for 30 min. The aqueous supernatant was collected before being layered onto a 20% sucrose pad and ultracentrifuged (at 100,000× *g*) for 2.5 h. The precipitate of the virus particles was suspended in 0.01 M sodium phosphate buffer containing 0.01 M MgCl_2_ (PB-Mg) and centrifuged at (7000× *g*) for 5 min. The supernatant was fractionated through a 10% to 40% sucrose gradient by centrifugation (78,000× *g*) for 2 h. Fractions were collected and re-suspended in 0.01 M sodium phosphate buffer. Isolated viral particles were negatively stained with 2% uranyl acetate. The particles were observed under a transmission electron microscope (JEM-1230, JEOL, Japan). The extracted viral particles were analyzed by 10% sodium dodecyl sulphate-polyacrylamide gel electrophoresis (SDS-PAGE) and stained with Coomassie brilliant blue (Sangon Biotech, Shanghai, China).

### 2.5. DNA Isolation and PCR Amplification

Total genomic DNA was extracted from the leaves of *Solanum tuberosum* seedlings using the cetyltrimethylammonium bromide (CTAB) method as described by Sambrook et al. [25]. The candidate plant DNA fragment was amplified using primer pairs that were designed based on the virus-related sequences. The polymerase chain reaction (PCR) product was electrophoresed on 1.2% agarose gel and stained with ethidium bromide (Sangon Biotech, Shanghai, China).

## 3. Results

### 3.1. Three dsRNA Segments in Strain HZ-1 Are Associated with a Mycovirus

**Figure 1:**
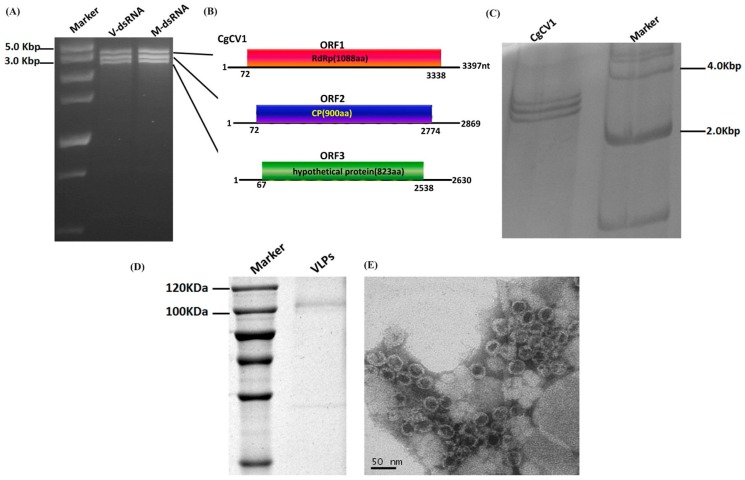
Agarose gel electrophoresis of double-stranded RNA (dsRNA) isolated from *Colletotrichum gloeosporioides* strain HZ-1, the genome organization of Colletotrichum gloeosprioides chrysovirus 1 (CgCV1), and analysis of isometric virus-like particles (VLPs) of CgCV1. (**A**) dsRNA segments were electrophoresed on a 2% agarose gel stained with ethidium bromide, with the size estimated using a DNA size marker. V-dsRNA and M-dsRNA indicate the dsRNAs extracted from the VLPs and mycelium mass, respectively; (**B**) Diagrammatic representation of the putative genome organization of CgCV1, consisting of three dsRNAs. Each dsRNA segment potentially encodes the RNA-dependent RNA polymerase (RdRp) (dsRNA1), the coat protein (CP) (dsRNA2), and the putative protease (dsRNA3), which are represented by rectangular boxes with different colors. The initiation and termination codons of the respective open reading frames (ORFs) are indicated by the numbers under the solid lines; (**C**) Silver nitrate-stained 12% acrylamide gel showing dsRNAs from CgCV1; (**D**) 10% sodium dodecyl sulphate-polyacrylamide gel electrophoresis (SDS-PAGE) analysis of the protein, which was indicative of a capsid protein of CgCV1 extracted from the virus-infected fungal strain HZ-1 (lane 2). The molecular weight of the protein band was estimated by the protein marker in lane 1; (**E**) Isometric VLPs were observed by transmission electron microscopy (TEM) after being negatively stained with 2% uranyl acetate. Scale bar = 50 nm.

When screening for the presence of dsRNAs in *C. gloeosporioides* isolates, we found that strain HZ-1 contained three virus-like dsRNAs (ranging in size from 2.5 kbp to 3.5 kbp), which are referred to as ‘dsRNA1 to 3’ according to their decreasing size (Figure 1A). These dsRNA extracts were confirmed to be dsRNA in nature, given their resistance to DNase I and S1 nuclease digestion. Purification of VLPs from mycelia of strain HZ-1 was conducted to investigate these dsRNAs. Spherical VLPs (approximately 40 nm in diameter) with the same three dsRNAs were examined by transmission electron microscopy (TEM) (Figure 1E). Consequently, these three dsRNAs were confirmed as the genomic segments of a novel mycovirus.

### 3.2. Molecular Cloning of dsRNAs Associated with Strain HZ-1

We employed cDNA library synthesis and terminal ligation mediated amplification to clone the complete sequences of these dsRNAs. The sequences of dsRNA1, 2, and 3 were deposited in Genbank under the accession numbers KT581957, KT581958, and KT581959, respectively. BLASTp searches, using the deduced amino acid sequences of dsRNA1, showed that it putatively encoded a viral RdRp displaying a high degree of sequence similarity to the RdRps encoded by viruses in the genus *Chrysovirus*. We presume these three dsRNAs to be the genome of a chrysovirus, named Colletotrichum gloeosporioides chrysovirus 1 (CgCV1). The schematic representation of the genome of this virus is presented in Figure 1B.

Notwithstanding, as most chrysoviruses have been reported to harbor four dsRNA segments of genomes, we repeated our cDNA cloning experiments in order to exclude the possibility that CgCV1 has the fourth genomic segment. All of the cDNA clones constructed from both the individual segment and total dsRNA segments that were extracted from virions, as well as fungal mycelia, have been matched to the original sequenced dsRNA segments. No additional virus-related fragments could be found. Further, electrophoretic examination of the dsRNA samples by PAGE was performed and showed that no additional fragments could be detected (Figure 1C). During a series of subcultures, the dsRNAs banding pattern of strain HZ-1 was shown to be constant. The results described above indicated that these dsRNAs, extracted from strain HZ-1, constitute the genome of a novel chrysovirus harboring only three genome segments.

### 3.3. Molecular Analysis of the Three dsRNA Segments

**Figure 2:**
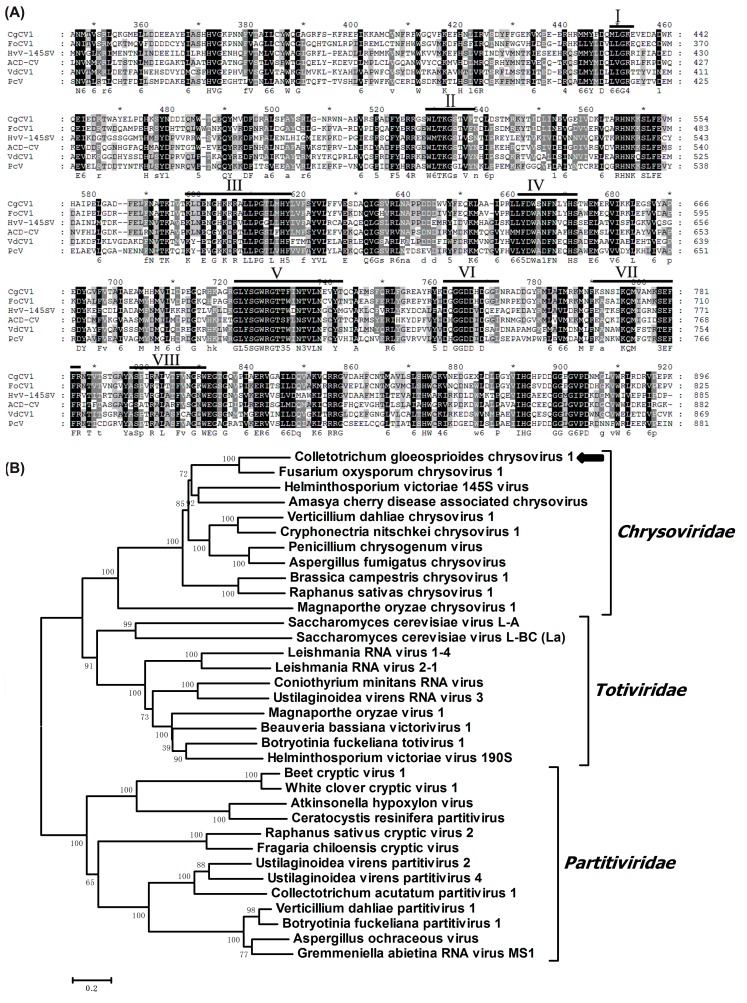
Alignment of amino acid sequences of viral RNA-dependent RNA polymerase (RdRp), and phylogenetic analysis of virus CgCV1. (**A**) Multiple alignments of putative RdRps of CgCV1 and other chrysoviruses sharing significant sequence similarities with CgCV1. The alignment was performed by CLUSTAL X and highlighted with the GeneDoc application. The conserved motifs of RdRp in dsRNA viruses are indicated by Roman numerals; (**B**) Phylogenetic analysis of the viral RdRp amino acid sequences of CgCV1. The phylogenic tree was constructed using the neighbor-joining method (1000 bootstrap replicates) in MEGA 6. Members in the families of *Totiviridae*, *Partitiviridae*, and *Chrysoviridae* were selected to conduct the multiple amino acid sequences alignment and phylogenic tree construction. These virus names and GenBank accession numbers are listed as follows: Amasya cherry disease-associated chrysovirus (CAH03664.1), Aspergillus fumigatus chrysovirus (CAX48749.1), Aspergillus ochraceous virus (EU118277), Atkinsonella hypoxylon virus (L39125.1), Beauveria bassiana RNA virus 1 (CCC42235.1), Beet cryptic virus 1 (EU489061.1), Botryotinia fuckeliana partitivirus 1(AM491609), Botryotinia fuckeliana totivirus 1 (AM491608), Brassica campestris chrysovirus 1(AKU48197.1), Ceratocystis resinifera partitivirus (AY603052.1), Colletotrichum acutatum RNA virus 1 (AGL42312.1), Coniothyrium minitans RNA virus (AF527633), Cryphonectria nitschkei chrysovirus 1(ACT79256.1), Fragaria chiloensis cryptic virus (AAZ06131.2), Fusarium oxysporum chrysovirus 1 (ABQ53134.1), Gremmeniella abietina RNA virus MS1 (AY089993), Helminthosporium victoriae 145 S virus (YP_052858.1), Helminthosporium victoriae virus 190 S (U41345), Leishmania RNA virus 1–4 (NP_619653.1), Leishmania RNA virus 2–1 (U32108), Magnaporthe oryzae virus 1 (YP_122352.1), Magnaporthe oryzae chrysovirus 1 (BAJ15133.1), Penicillium chrysogenum virus (YP_392482.1), Raphanus sativas chrysovirus 1(AFE83590.1), Raphanus sativus cryptic virus 2 (ABB04855.1), Saccharomyces cerevisiae virus L-A (AAA50321.1), Saccharomyces cerevisiae virus L-BC (La) (NP_042581.1), Ustilaginoidea virens partitivirus 2 (AGR45851), Ustilaginoidea virens partitivirus 4 (AGJ03719), Ustilaginoidea virens RNA virus 3 (AHH25155.1), Verticillium dahliae chrysovirus 1 (ADG21213.1), Verticillium dahliae partitivirus 1 (AGI52210.1), and White clover cryptic virus 1 (AY705784.1).

The dsRNA1 of CgCV1 was 3397 nt in length and contained an open reading frame (ORF), initiating at the nucleotide in position 72 and terminating at position 3338. The ORF was predicted to encode a 1088 amino acid (aa) protein with a calculated molecular mass of 126 kDa. A BLASTp search showed that the 126 kDa protein has a maximum identity of 63% (*E*-value: 0; query cover: 78%) in a deduced aa sequence compared to that encoded by Fusarium oxysporum chrysovirus 1 (FoCV1), followed by other chrysoviruses, including Helminthosporium victoriae 145S virus (HvV145S) (*E*-value: 0; query cover: 98%; identity: 39%), Verticillium dahliae chrysovirus 1 (VdCV1) (*E*-value: 0; query cover: 96%; identity: 36%), Penicillium chrysogenum virus (PcV) (*E*-value: 0; query cover: 98%; identity: 38%), and other related viruses. Multiple alignments and conserved domain database (CDD) searches confirmed that the dsRNA1-encoded protein contained eight conserved motifs characteristic of RdRps of dsRNA viruses infecting lower eukaryotes [26] (Figure 2A). In addition, a ‘phytoreovirus S7 domain’ that is widely distributed in *Chrysoviridae*, *Totiviridae*, *Reoviridae*, and *Endornaviridae* was also found in the N-terminal region of the RdRp encoded by dsRNA1 [6,7,27]. Phylogenetic analysis, based on the alignment of the deduced aa sequence of the RdRps in CgCV1, and other selected viruses in the family *Chrysoviridae*, showed that the CgCV1-encoded RdRp was grouped with those RdRps that were encoded by viruses in the genus *Chrysovirus* (Figure 2B).

The dsRNA2 of CgCV1 was determined to be 2869 bp in length and with a single large ORF from nt positions 72 to 2774, potentially encoding a protein of 101.7 kDa composed of 900 aa. BLASTp searches, using the deduced aa sequences of dsRNA2 ORF, showed high similarity to the CPs of other known and tentative members of the family *Chrysoviridae*, with HvV145S CP being the best matching (*E*-value: 7 × 10^−107^; query cover: 72%; identity: 33%). The homology search results suggested that the dsRNA2 might be a CP-encoding segment of CgCV1. Coincidentally, a single major protein band of approximately 100 kDa was detected in the VLPs by SDS-PAGE analysis (Figure 1D), which can serve as proof of the existence of the viral CP of CgCV1. Notably, the region that shared high sequence similarities among the CPs (of these related viruses) was mainly limited to the CPs N-terminal halves of 560 aa residues. A CP-based phylogenetic tree was generated and showed similar topology to that constructed from the RdRp alignment (Figure S1).

The dsRNA3 of CgCV1 was composed of 2630 nt, within which a single large ORF (starting at nt 67 and terminating at nt 2538) was found. The single ORF potentially encoded an 823 aa protein of 92 kDa. A homology search using BLASTp revealed that a number of sequences have similarities to the dsRNA3-encoded protein. The most closely related hit was the HvV145S-protein 3 (*E*-value: 5 × 10^−125^; query cover: 99%; identity: 32%). In addition, an uncharacterized protein of *Solanum tuberosum* (Genbank accession: XP_006340960), and two putative proteins of *Medicago truncatula* (Genbank accessions: XP_003616855 and XP_003616852, respectively) encoded by two different genes (ORFs of 1300 bp and 1608 bp, respectively) in chromosome 5 of *M. truncatula* were also found to have similarities with the CgCV1-dsRNA3-encoded protein. Multiple alignments of the dsRNA3-encoded protein and other homologous proteins of members of the family *Chrysoviridae* revealed that the dsRNA3-encoded protein of CgCV1 also contained the motif PGDG (K/S) CGXHA (Figure S2). This motif, as previously illustrated by Covelli et al. [28], is encoded by most of the known chrysoviruses and is one of the four motifs constituting the conserved core of a putative ovarian tumor (OTU) gene-like superfamily [29,30]. Though the OTU peptidase might be widespread in other viruses, even in eukaryotes and bacteria, its functions are yet to be definitively ascertained [31].

### 3.4. 5′- and 3′-Untranslated Regions (UTRs)

**Figure 3:**
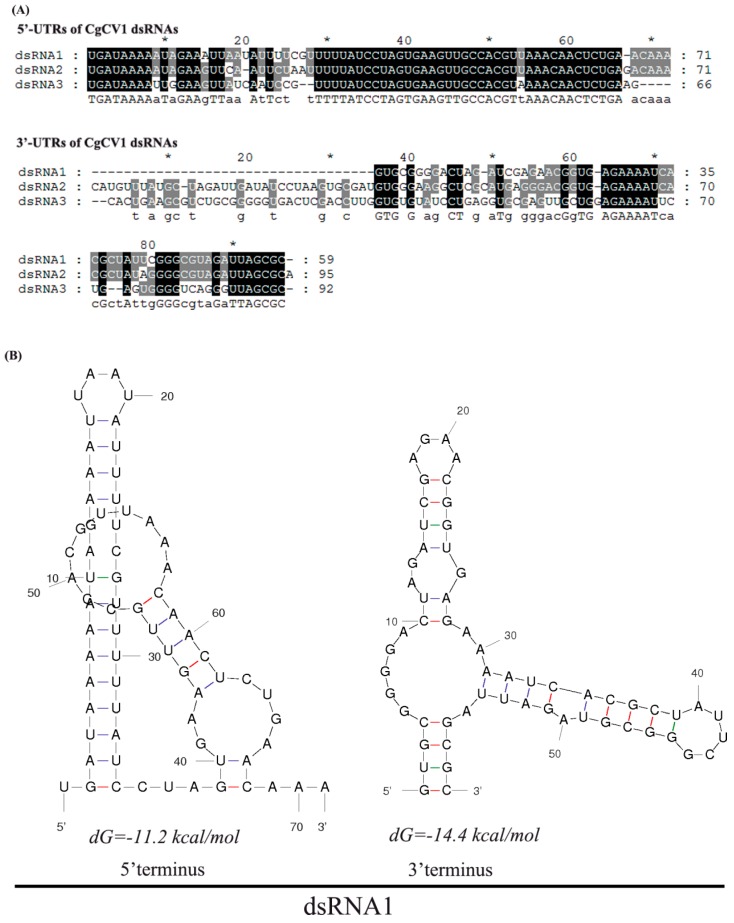
Terminal sequence comparison and secondary structure prediction of the coding strand of CgCV1 dsRNAs. (**A**) Conserved regions among the three dsRNAs in the 5′ or 3′ terminus are indicated with black shading; (**B**) secondary structures in the 5′ and 3′ terminal regions of CgCV1 dsRNA1 were predicted by the Mfold program with the lowest energies.

The UTRs for CgCV1 dsRNA1, 2, and 3, were 71, 71, and 66 bp in length (respectively, at their 5′-end) and 59, 95, and 92 bp in length (respectively, at their 3′-end). Multiple alignment of 5′ UTRs of the three CgCV1 dsRNAs indicated that conserved regions with high sequence similarity could be observed (Figure 3A). The 5′-termini (position 1–14) contained a conserved sequence stretch of UGAUAAAAA(/U)UA(/G)GAA, among which the stretch of sequence GAUAAAA was shared by other chrysoviruses, such as PcV, Aspergillus fumigatus chrysovirus (AfuCV), VdCV, and HvV145S, but not with Amasya cherry disease-associated chrysovirus (ACDACV), Cryphonectria nitschkei chrysovirus 1 (CnV-1), and Botryosphaeria dothidea chrysovirus 1 (BdCV1) dsRNAs. This can be observed from another alignment using the 5′ UTRs of other reported chrysoviruses. In addition, with the exception of nucleotide position 54, a second conserved stretch (from 29 nt to 66 nt) was also found. The 3′ UTRs of the three CgCV1 dsRNAs also have some degree of sequence similarity, containing identical sequence stretches (UUAGCGC) at the 3′-termini and stretches (AGAAAAU) at the internal region, respectively. However, the similarity of the 3′ UTRs between CgCV1 and other reported chrysoviruses was relatively low. The 5′ and 3′ UTRs, as illustrated for dsRNA1 (Figure 3B), were predicted to fold into stable stem-loop structures.

### 3.5. Searching for CgCV1-Like Sequences in Extant RNA Viral Sequences and Eukaryotic Nuclear Genomes

In another independent experiment, we also searched the eukaryotic genomes in NCBI databases by tBLASTn analysis for the presence of CgCV1-like sequences (Table S1). Using an amino acid sequence of each genome segment of CgCV1 as a query, we found several chrysovirus like-contigs conspicuously matching to plants (such as the *Noccaea caerulescens*, *Artemisia annua* (sweet wormwood), *Allium fistulosum*, *Vigna radiate*, *Colobanthus quitensis*, and *Camellia taliensis*) in the TSA library databases. Among these, contigs of *Noccaea caerulescens* and *Artemisia annua* were all related to CgCV1 dsRNA1, 2, and 3. For NCBI expressed sequence tag database (ESTdb) searches, no significant hits for CgCV1 dsRNA2-encoded protein sequences could be found in the available ESTdb. However, sequences of *Zinnia violacea* and *Dactylis glomerata* (in the EST library) were found to have significant identities with the CgCV1 dsRNA1 and 3. These contigs were conversely confirmed (by reverse BLAST analysis against the Genbank nucleotide (nr/nt) database) to be closely related to the corresponding regions of putative proteins encoded by chrysoviruses. Distinctly, some sequences from plant genomes were closely related to the CgCV1 dsRNA3 protein. These sequences included four putative proteins from *Medicago truncatula*, which were named MTgP1 to MTgP4 (*M. truncatula* genomic hypothetical protein MTR 5g084940, *M. truncatula* chromosome 5 clone mth2-75o19, *M. truncatula* hypothetical protein MTR 5g084970, and *M. truncatula* chromosome 5 clone mte1-85h10), and two proteins from *S. tuberosum* (LOC102579694) and *Trifolium pretense*, respectively. Thus, endogenization of chrysovirus sequences may have occurred in these plants.

**Figure 4:**
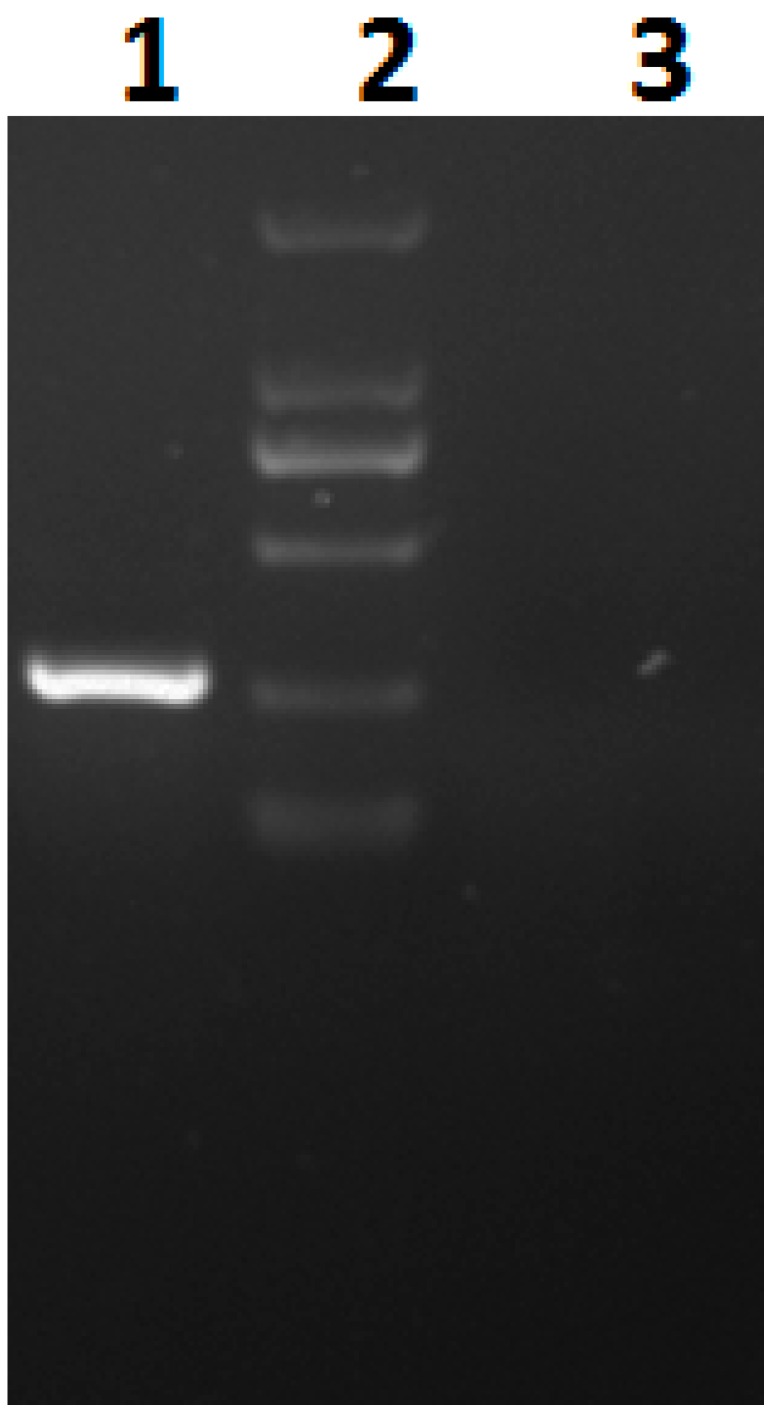
Polymerase chain reaction (PCR) amplification of the total DNA of *S. tuberosum* using a primer pair specific to CgCV1-dsRNA3-like sequence. From left to right: DNA template extracted from *S. tuberosum* (lane 1), Marker in size rank of 100 bp, 250 bp, 500 bp, 750 bp, 1000 bp, 2000 bp (lane 2), DNA template extracted from control plant (lane 3).

In order to confirm the presence of CgCV1-dsRNA3-like sequences in plant chromosomes, we used PCR amplification with primer pairs corresponding to the CgCV1-dsRNA3-like *S. tuberosum* sequences. An amplicon of the anticipated size was obtained from *S. tuberosum* but not from the control plant of *Solanum lycopersicum* (Figure 4). Sequencing of the amplicon showed that it was identical to the relevant region of the *S. tuberosum* genome, thus indicating that the CgCV1-dsRNA3- like sequence was indeed present in the *S. tuberosum* genome rather than contaminants during the *S. tuberosum* genome sequencing.

## 4. Discussion

As reported in this paper, three dsRNA segments isolated from *C. gloeosporioides* strain HZ-1 were identified. Sequence analysis provided evidence that each of the identified dsRNA segments contained a major ORF, putatively encoding an RdRp, major CP, and protease, respectively (homologous to segments of the genus *Chrysovirus*). We deduced that the three dsRNA segments constitute a novel chrysovirus named ‘CgCV1’, regardless of their trisegmented genome. Several observations may verify the trisegmented nature of the chrysovirus CgCV1. The three segments shared conserved terminal sequences at both ends, especially the terminal sequence stretch (GAUAAAA), which existed in all three dsRNA segments and was shared by other chrysoviruses. The presence of conserved terminal sequences is commonly observed in genome segments of multipartite viruses, which have been considered important for virus transcription, replication, and packaging [32]. Purified preparations of VLPs (approximately 40 nm in diameter) also contained all three dsRNA segments. Only dsRNA1 encoded an RdRp, indicating that the other two dsRNAs might depend on this RdRp for replication and together with the dsRNA1 form the genome of CgCV1 rather than a derivation from multiple independent mycoviruses. Furthermore, the dsRNA pattern of CgCV1 has remained unaltered (throughout two years of culture analysis) since the first extraction of the dsRNAs. These findings led us to establish that the three dsRNA segments represent the genome of a novel virus. Phylogenetic analyses of the RdRp of CgCV1 strongly suggested that this virus is most closely related to the typical chrysovirus FoCV1. Based on genome segment size, sequence and structural features of their conserved 5′ UTRs, amino acid sequence similarity of RdRp, the presence of specific motifs, and virion size, we propose that CgCV1 is a new species of the genus *Chrysovirus*. To our knowledge, this is the first report of a chrysovirus existing within *C. gloeosporioides*.

Notably, CgCV1 described in this study has only three dsRNA genome segments, distinguishable from previous studies that reported that most chrysoviruses have four dsRNA genomic segments [6,16,28]. However, some none-quadripartite chrysoviruses have also been found not only in fungi but also in plants. For instance, the Fusarium graminearum mycovirus-China 9, a chrysovirus isolated from phytopathogenic fungus *Fusarium graminearum*, has a genome of five dsRNA segments [33]. The Magnaporthe oryzae chrysoviruses 1-A and -B were also reported to be pentapartite chrysoviruses infecting the rice blast fungus *Magnaporthe oryzae* [34,35]. In addition, a trisegmented chrysovirus named Raphanus sativus chrysovirus was isolated from the radish, *Raphanus sativus* [7]. These reports mean that the numbers of genomic segments for the chrysoviruses were not always constant, and some genome segments might be dispensable for the life cycle of the chrysoviruses. Significantly, the dsRNA3-encoding protein of CgCV1 was similar to the dsRNA4 components of other chrysoviruses, such as PcV, AfuCV, and VdCV1, as well as the dsRNA3 products of other known and tentative chrysoviruses sequenced thus far.

CgCV1-like sequences in plant genomes provided some insights into the evolution of the chrysoviruses. Some protein sequences that shared similarities to the CgCV1 dsRNA3 have been identified in the genome of *M. truncatula* and *S. tuberosum*. Furthermore, tBLASTn searches against the NCBI TSA database, using each segment of CgCV1, showed CgCV1-like contigs in the plants of *Noccaea caerulescens*, *Artemisia annua*, *Allium fistulosum*, *Vigna radiate*, *Colobanthus quitensis*, and *Camellia taliensis*. Contigs relating to CgCV1 dsRNA1, 2, and 3 were detected in clones from *N. caerulescens* and *A. annua*, thereby suggesting that a CgCV1-like chrysovirus may have infected these hosts. Some unidentified chrysoviruses might exist in a wide range of host organisms, as in other non-retroviral RNA viruses [7,26,27,36]. It remains to be examined whether the CgCV1-like sequences, identified from the NCBI TSA/EST databases, were derived from the genomic RNA of infecting novel viruses or from endophytic fungi (as transcripts or viruses) that might be contaminated with the plant materials, or even from endogenous transcripts corresponding to integrated host chromosomal virus-like sequences.

## Supplementary Materials

The following are available online at www.mdpi.com/1999-4915/8/10/268/s1, Figure S1: Phylogenetic analysis of the viral capsid protein (CP) amino acid sequences of Colletotrichum gloeosprioides chrysovirus 1 (CgCV1), Figure S2: Multiple alignment of the amino acid sequences in the C-terminal region between the dsRNA3 of CgCV1 and other comparable regions of similar chrysoviruses, Table S1: CgCV1-like sequences in the NCBI database.
