# Supplementary material for: Molecular Characterization of a Trisegmented Mycovirus from the Plant Pathogenic Fungus Colletotrichum gloeosporioides
Source: Viruses. 2016 Sep 27;8(10):268. doi: 10.3390/v8100268 (PMC5086604; doi:10.3390/v8100268)
Supplement: Supplementary file 1 [file viruses-08-00268-s001.pdf]

Supplementary Materials: Molecular Characterization of a Trisegmented Mycovirus from the Plant Pathogenic Fungus *Colletotrichum gloeosporioides*

Jie Zhong, Xi Dan Pang, Hong Jian Zhu, Bi Da Gao, Wen Kun Huang, and Qian Zhou

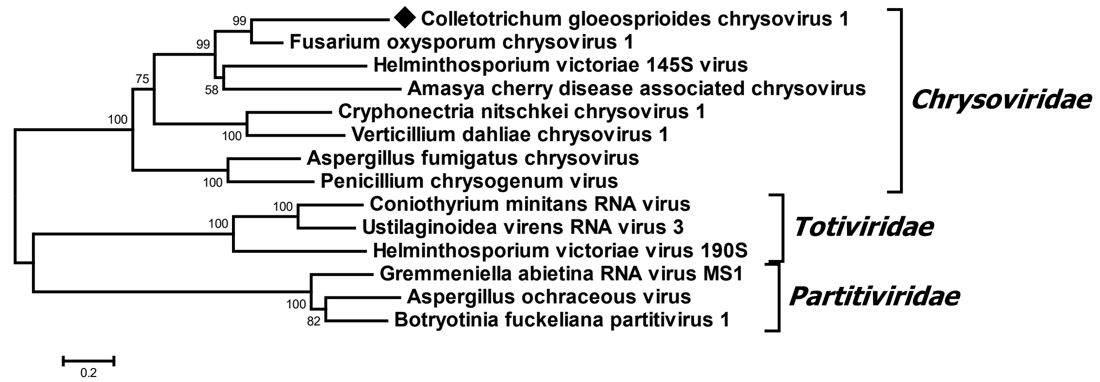

Figure S1. Phylogenetic analysis of the viral capsid protein (CP) amino acid sequences of Colletotrichum gloeosprioides chrysovirus 1 (CgCV1). The phylogenetic tree was generated using the neighbor-joining method in MEGA6 [24] (with a 1000-replicate bootstrap search). The values on the nodes refer to the percentage of bootstrap replicates supporting the branch. Respective GenBank accession numbers of these analyzed viruses are shown as follows: Amasya cherry disease-associated chrysovirus (YP_001531162.1), Aspergillus fumigatus chrysovirus (CAX48751.2), Aspergillus ochraceous virus (ABV30676.1), Botryotinia fuckeliana partitivirus 1 (CAM33267.1), Coniothyrium minitans RNA virus (YP_392466.1), Cryphonectria nitschkei chrysovirus 1 (ACT79252.1), Fusarium oxysporum chrysovirus 1 (ABQ58816.1), Gremmeniella abietina RNA virus MS1 (NP_659028.1), Helminthosporium victoriae 145S virus (YP_052859.1), Helminthosporium victoriae virus 190S (NP_619669.2), Penicillium chrysogenum virus (YP_392483.1), Raphanus sativas chrysovirus 1 (AFE83591.1), Ustilaginoidea virens RNA virus 3 (YP_009004155.1), and Verticillium dahliae chrysovirus 1 (ADG21214.1).

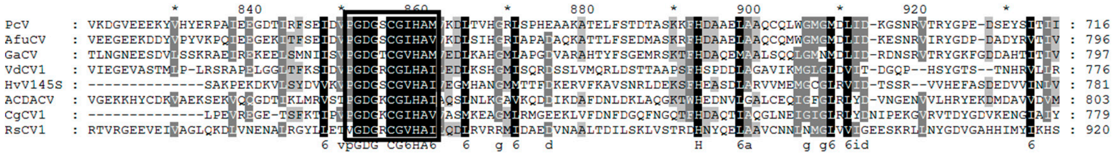

Figure S2. Multiple alignment of the amino acid sequences in the C-terminal region between the dsRNA3 of CgCV1 and other comparable regions of similar chrysoviruses. The alignment was made using CLUSTAL X [23]. The conserved motif is indicated by the black box. The full names and GenBank accession numbers of these viruses are listed as follows: PcV, Penicillium chrysogenum virus (FN178515); AfuCV, Aspergillus fumigatus chrysovirus (FN178515); GaCV, Grapevine chrysovirus (AFX73020.1); VdCV1, Verticillium dahliae chrysovirus 1 (HM004070); HvV145S, Helminothosporium victoriae 145S virus (NC005980); ACDACV, Amasya cherry disease-associated chrysovirus (AJ781164); and RsCV1, Raphanus sativas chrysovirus 1 (AFE83592.1).

Table S1. CgCV1-like sequences in the NCBI database.

Database	Species	Contig/Singleton	dsRNA1			dsRNA2			dsRNA3		
			Coverage (%)	Identity (%)	E-Value	Coverage (%)	Identity (%)	E-Value	Coverage (%)	Identity (%)	E-value
TSA	*Noccaea caerulescens*	isotig06317_isogroup02885 (GASZ01006339.1)	67	37	7×10^{-155}						
	Noccaea caerulescens	Nc_isotig05844_isogroup02412 (GASZ01005866.1)				53	26	1×10^{-44}			
	Noccaea caerulescens	Nc_isotig05933_isogroup02501 (GASZ01005955.1)							57	30	7×10^{-47}
	Camellia taliensis	CtaTrans37938 (GCRZ01034361.1)	65	29	7×10^{-83}						
	Camellia taliensis	CtaTrans13937 (GCRZ01012637.1)	19	28	2×10^{-15}						
	Camellia taliensis	CtaTrans29091 (GCRZ01026355.1)							38	27	5×10^{-10}
	Artemisia annua	Artemis Contig26286 (EZ166880.1)							73	25	4×10^{-39}
	Artemisia annua	Artemis Contig6582. Aml (EZ193025.1)							59	25	2×10^{-25}
	Artemisia annua	Artemis Contig3687. Aylt (EZ206676.1)							47	25	8×10^{-24}
	Artemisia annua	Madagascar Contig12667. Blt (EZ326121.1)							57	24	2×10^{-23}
	Artemisia annua	Madagascar Contig9777. Mt (EZ283390.1)				8	33	1×10^{-4}			
	Artemisia annua	Artemis Contig7017. Aylt (EZ210006.1)				6	36	4×10^{-4}			
	Artemisia annua	Artemis Contig11776. Aml (EZ198219.1)				16	27	6×10^{-10}			
	Artemisia annua	Artemis Contig24510 (EZ165104.1)				9	35	6×10^{-7}			
	Artemisia annua	Uganda Contig20290 (EZ403795.1)				7	35	7×10^{-7}			
	Artemisia annua	ArtemisContig3870. Aml (EZ190313.1)	39	43	6×10^{-11}						
	Artemisia annua	Artemis Contig25044 (EZ165638.1)	23	50	6×10^{-71}						
	Artemisia annua	Artemis Contig4906. Aylt (EZ207895.1)	22	36	2×10^{-41}						
	Artemisia annua	Artemis Contig4484. Amlt (EZ237814.1)	16	43	8×10^{-38}						
	Artemisia annua	Artemis Contig24942 (EZ165536.1)	17	36	1×10^{-34}						
	Artemisia annua	Artemis Contig 9161. Aml (EZ195604.1)	18	37	1×10^{-28}						
	Artemisia annua	Artemis Contig7563. Aylt (EZ210552.1)	18	37	4×10^{-28}						
	Artemisia annua	Artemis Contig1636. Amlt (EZ234966.1)	18	36	5×10^{-27}						
	Artemisia annua	Uganda Contig17865 (EZ401370.1)	9	52	1×10^{-25}						
	Artemisia annua	Contig1233. Amlt (EZ234563.1)	16	37	2×10^{-24}						
	Artemisia annua	Contig1198. Amlt (EZ234528.1)	7	58	1×10^{-22}						
	Artemisia annua	Contig2871. Aml (EZ189314.1)	15	33	1×10^{-22}						
	Artemisia annua	Madagascar Contig18240. Mt (EZ29185 3.1)	10	42	2×10^{-20}						
	Artemisia annua	Uganda Contig5658. Uht (EZ358497.1)	7	49	5×10^{-19}						
	Artemisia annua	Artemis Contig10169 (EZ150763.1)	14	31	9×10^{-19}						
	Artemisia annua	Madagascar Contig6103 (EZ263000.1)	5	63	7×10^{-18}						
	Artemisia annua	Artemis Contig6641 (EZ147235.1)	10	44	3×10^{-17}						
	Artemisia annua	Artemis Contig6413. Aylt (EZ209402.1)	6	54	5×10^{-17}						
	Artemisia annua	MadagascarContig19552. Mt (EZ293165.1)	12	35	1×10^{-15}						
	Artemisia annua	Contig7536 (EZ148130.1)	15	31	2×10^{-14}						
	Artemisia annua	Artemis Contig5462. Amlt (EZ238792.1)	10	32	1×10^{-13}						
	Artemisia annua	Uganda Contig21105 (EZ404610.1)	12	36	1×10^{-12}						
	Artemisia annua	Artemis Contig2500. Ac (EZ179024.1)	11	32	3×10^{-12}						

	Artemisia annua	Artemis Contig947. Ac (EZ177471.1)	6	55	5×10^{-12}						
	Artemisia annua	Madagascar Contig7630. Mt (EZ281243.1)	9	34	7×10^{-11}						
	Artemisia annua	Artemis Contig6864. Aylt (EZ209853.1)	5	48	4×10^{-10}						
	Artemisia annua	Artemis Contig1606. Amlt (EZ234936.1)	10	32	1×10^{-7}						
	Artemisia annua	Uganda Contig3311. Ulm (EZ369860.1)	7	37	2×10^{-7}						
	Artemisia annua	Uganda Contig2972. Ulm (EZ369521.1)	6	42	4×10^{-7}						
	Artemisia annua	Artemis Contig1982. Aylt (EZ204971.1)	7	36	1×10^{-6}						
	Artemisia annua	Artemis Contig1184 (EZ141778.1)	5	40	2×10^{-6}						
	Allium fistulosum	contig: AFU_22548 (FX576273.1)	17	31	1×10^{-21}						
	Allium fistulosum	contig: AFU_01016 (FX554741.1)				52	26	5×10^{-38}			
	Allium fistulosum	contig: AFU_00430 (FX554155.1)							48	31	2×10^{-43}
	Bemisia tabaci	comp13683_c0_seq1 (GARP01025880.1)	39	21	3×10^{-5}						
	Caligus rogercresseyi	tsa-crog-ngs-11124173 (GAZX01040384.1)	47	23	2×10^{-10}						
	Caligus rogercresseyi	tsa-crog-ngs-11124172 (GAZX01040383.1)	47	23	2×10^{-10}						
	Caligus rogercresseyi	tsa-crog-ngs-11124002 (GAZX01040226.1)	45	23	3×10^{-8}						
	Humulus lupulus var	contig: comp13164 2_c0_seq1 (LA332420.1)	27	26	3×10^{-10}						
	Grylloblatta bifratrilecta	s13623_L_120298_0 (GAWP01013600.1)	15	31	4×10^{-10}						
	Grylloblatta bifratrilecta	s17247_L_150202_0 (GAWP01017218.1)	32	24	2×10^{-7}						
	Grylloblatta bifratrilecta	s7948_L_90492_0 (GAWP01007931.1)	14	30	4×10^{-8}						
	Phakopsora pachyrhizi	Thai1Pp_contig01637 (GACM01002541.1)	25	24	1×10^{-7}						
	Phakopsora pachyrhizi	Thai1Pp_contig04037 (GACM01003506.)	28	25	4×10^{-6}						
	Uromyces appendiculatus	Ua_contig01686 (GACI01004215.1)	19	28	9×10^{-7}						
	Teleopsis dalmanni	Td_comp6756_c0_seq1 (GBBP01176362.1)	20	26	1×10^{-6}						
	Lepeophtheirus salmonis	lsal-pac-6261972 (JP332015.1)	18	25	7×10^{-6}						
	Mengenilla moldrzyki	contig03151 (JP089782.1)	27	22	8×10^{-06}						
	Acanthosoma haemorrhoidale	C369386 (GAUV01086077.1)	6	38	1×10^{-5}						
	Vigna radiata	Vr16063_c0 (GBXO01026945.1)				21	33	8×10^{-18}			
	Colobanthus quitensis	Colobanthus_quitensis_contig_31970_1 (GCIB01032448.1)				18	30	8×10^{-12}			
EST	*Zinnia violacea*	AU307126 cDNA cloneZ16843 (AU307126.1)	16	49	4×10^{-45}						
	Zinnia violacea	FM881064 SSHSTS0 cDNA clone SSHSTS0 6xxxA02F2 (FM881064.1)							12	31	5×10^{-5}
	Zinnia violacea	FM881063 SSHSTS0 cDNA clone SSHSTS06 xxxA02F1 (FM881063.1)							12	31	5×10^{-5}
	Zinnia violacea	AU307096 cDNA clone Z16810 (AU 307096.1)							28	23	2×10^{-8}
	Dactylis glomerata	BG04060A1C03.f1 BG04- primary and normalized libraries (HO148595.1)	11	46	1×10^{-30}						
	Dactylis glomerata	BG04058A1B02.r1 BG04-cDNA (HO1476 84.1)							16	29	2×10^{-7}
	Lepeophtheirus salmonis	EST_lsal_af_785797 lsalaf whole Lepeophtheirus salmonis (Pacific) cDNA clone lsal_af_1_85_rev 5′	18	25	5×10^{-6}						
	Artemisia annua	CATA453.fwd CATA A Tanzanian, cDNA cloneCATA453 5′ (EY047151.1)							17	29	2×10^{-6}

NR	*Solanum tuberosum*	Solanum tuberosum uncharacterized LOC102579694 (LOC102579694) (XM_006340898.1)	73	24	5×10^{-37}
	Medicago truncatula	Medicago truncatula hypothetical protein (MTR_5g084970) (XM_003616807.1)	63	25	5×10^{-28}
	Medicago truncatula	Medicago truncatula chromosome 5 clone mth2-75o19 (CT573055.2)	66	25	9×10^{-27}
	Medicago truncatula	Medicago truncatula chromosome 5 clone mte1-85h10 (CR936947.2)	66	25	9×10^{-27}
	Medicago truncatula	Medicago truncatula hypothetical protein (MTR_5g084940) (XM_003616804.1)	48	24	2×10^{-19}
	Trifolium pratense	Trifolium pratense genome assembly redclover, chromos ome:chr4 (LN846352.1)	39	28	1×10^{-21}
HTGS	*Solanum tuberosum*	Solanum tuberosum strain Diploid genotype RH89-039-16 chromosome 9 clone RH204J10, (AC239340.1)	73	24	2×10^{-37}
chromoso me	*Solanum tuberosum*	Solanum tuberosum cultivar DM 1-3 516 R44 unplaced genomic scaffold, SolTub_3.0 scf00020, whole genome shotgun sequence (NW_006238946.1)	73	24	3×10^{-36}
	Medicago truncatula	Medicago truncatula chromosome 5 (NC_016411.1)	66	25	3×10^{-26}

Note: these similar sequences were collected by tBLASTn searches against the NCBI databases including nucleotide collection [nr/nt], high-throughput genomic sequences [HTGS], non-human, non-mouse expressed sequence tags [EST], and transcriptome shotgun assembly [TSA].

Genome sequences of Colletotrichum gloeosprioides chrysovirus 1 (CgCV1):

>dsRNA1

TGATAAAAATAGAAATTAATATTTTCGTTTTTATCCTAGTGAAGTTGCCACGTTAAACAACTCT
GAACAAAATGTCGGGACGAATGACTAGAATGAGTCAAACTTCAAAGAAGAAAGTGGAGCA
AGCAGCGGCAAATATACAAGCGAGAGTGCAGTATTTTGGATCTGACAAAGGTAGGATAGAC
TTTACTGCTGAAGTTCTGTCTTCGATGGAAAGATACGAAAAGAGGAGGAGAGATAACTTCTT
TGCGATCGTTATGCCGTCAGGTTCTGGTAAAACTCAACTTTGTGACAAGTATGGATTTATCGA
CGTTGACAGATTGTGCACAATAGCGGAACACGAGTACCTGAATGACCTAAGGCTGGATGCT
ATCAACGGGAAAGATACCTGGGATGAGCACAACAAAGTGTGGTTCGAGTTGTTGAACGAA
ACTCTGGACCTGATGGAGTTCAAGGAGGACACGGTTATCATGGTGCAGAGTGAGGTAATAG
CGTTGGGCATAGGGGCACTGCCTCTGGCTGGGTTCATCTTAGATGATAAGACATATCAGATG
GTGTATAACCAGAAAGTCGAAAGAGGGAATAGAGCAGGTGCTCTGCTAATGGCTGAGAAC
AGAGCCCAGTTTCTGAGCAACACGAACATAGTGATGAAAACAAAGAAGATGGTGAGAGGA
TTTGAGATGATGGAAGCAAAGGTGATAGAGACTCTGAATGTGAATGAAATACCATGCGCTTG
CCCGCTAAAATTCTCATTGCGATATCCGGGATACGGTTACGGCCCTTCTGTTCCTGATTGGGTT
ACGAAAGGGGAGCGAAAGTACATGAATGTTGTGGAATTGTTGGACCTGCATAGGTCAGGGA
TGGTGCCCAAACAGTGCATGGATTACTTCATGAGGGACCCAGAAATTCCTGCTTCTTACGGA
TTCGGCGTGAGAGACAATGAGTGGTCACAGTTCTTCGCAAACATGAGGGCTTCTATTGGCTC
ACCTCAGAACTTCGACCCAAGGGATGATCTGGGGAAGGTGTTCCCATACAAATACGAAAAG
AACTTGACGAGGGCGAACATGACAGTGAGTAGATTACAGAAAGGCATGGAACTGCTTGAC
GATGAGGAGGCTTATGAGATAGCGTCACATCATGTGGGCAAGCCAAACAATTTTGTGACAG
CAATATTGTGTTACTGGCAAGGGGCAGGTAGATTCTCAAAGTTCAGAGAGTTCATCAAGAA
GGCAATGTGTGTAAACTTTAGATTCTGGGGACAGGTGTTCAAAGAGTTCCACAATTTAATAA
GAGTATCAGACTATTTCTTTGGAGAAAAAGTGRGCGAAGAGGAAAGGCATAGAATGATGTA
CTTCGACCAGATGTTGGGGAAGGAAGTGGAGGATGCGGACTGGAAGCAGGAGATTGAGGA
TAGAACTTGGGCATATGAATTGCCAGACCACAAGTCTTACAACGATGATATACAAATGTGGA
CACAAGAACAATACATGGTCGATTTCGACAGAGCACTATCTTTTGCGTATTCTAGGCTGGGA
AATAGGTGGAATGCGGAAGTGAGATCGTTTGCTGACTTCTACGAAAGGAGAGGGTCTTGGC
TAACAAAGGGGAGTACGGTGTTCACACAATTGGATAGCACAATGCGGAAGTACACAATAGA
CTTAATCAATGAGGTGGGCGAGATAGTGGACAAACTGACAGCCAGGCACAACAAGAAGTC
GCTGTTCGAAGTGATGCATGCCATACCAGAATTGGGTGCAGACTTTGAATTGTTCAACGCGA
CCAAGATTGTTACCAAGTTGGACGAAAACGGTCACAAGAAAAGGGCCCTCTTACCGGGCA
CGCTGATGCACTACCTGGTGTTTTCATATGTGTTGTTTTTTGTGGAGTCCGATGCTCAAATAGG
GTCGGTGAGGTTGAATGCACCACCTGATGATGACTGGGTATATTTCGAGCAAAAGCTGGCTG
CAATTCCCAGATTGCTATTCGACTGGTCCAACTTCAATCTGTACCACTCAACCTGGGAAATG
GAGAGGGTGATAAAGAAATTGGAGGGCAGTGTTTATGCACCTGAGGATTATGGTGTATTTGT
GACTGCGATAGCGGAGGCTATGCACCACATGGTGATAATAGATCCTGAAGGGCAGAGGCAT
AAGGCAGGGAGAGGGTTGTACTCGGGTTGGAGAGGAACAACTTTCATCAACACTGTGCTAA
ATGAGGTGTACACCCAATGTGCAGAGATGAGTTTTCAGAGATTGTTCGGAAGGGAGGCCTA
CCGATATAAAGATGGCGGTGGCGACGATCACGACGGTGGGTTGAACAGAGCAGACGATGG
GTACAGGATGTTGGCAATAATGAGGAAAATGAATTTCAAATCAAATTCCATAAAGCAGATGG
TTGCAATGAAAAGTGAGTTCTTTAGAAACACTGTGACATCGACAGGTGCTTACGCTAGTCCA
ATTAGGGCTCTAGTAACATTTGTGAATGGTAGGTGGGAAGGAACAGGACAGGTGCCGATAG
CCGAGAGAGTAGGAGCGATATTGGATCAAGTTGCGAAGGTACAAAGAAGAGGGGTGGACG
CACACTTCTGCAATACAATGGCAGTGTTGTCTCTTAGTCACTGGTGCAAGGTGAACGAGGAC
GGCGAGTKGTTAGACTTGCCCGGTTATGTAATACATGGCCATCCGGATGACGGAGGCTTTGG
AGTACCGGATAACATGGGACTGGTCTGGAGACTGAGGGACAGGGTGCCTGAGCCTAAGGTG
ATTGGGGAAGCGAGAGAAGTGCCGGGAACATTGGCAACTAGGGATTGGTTGTCAGTGCTTT
GCAGAGAAGTGAGGTCTTTGAACATTGATGTGGACATAGATGAAAAGAAAGTGATAGATAC
AGCGAAAAGCTCATTCGAGGCTTACAGTAGGTATGATTATACAGAAGTGTTGAAGTTCAGAA
CAGAAATAGTGGGCAAGGCGCCGGCTGTGGAACCTCGAAACAACGAGTCGGCCTGGGAAT

TCATGGTGGAATTCATGGCAATAGAAAAACACGATCTTAAAATTGGCAAACTC
ATGAGGTACCAGGAATTGATACCTTATATGACAGTTGGCGGAAAACCGATGACAAGACAGC
AACTGGGCGAAGCTATGGAGTTGGGTGTGAATACAGAAGTCGTAGACTTCAAAGGAGACGT
GTATTATAGGAGACTGATATCAGAGCCATTAGCAAAGGTAGTAACTGAGTTCTGCATGGAAG
CAATAGCCAGGGAGATATATAGTCTAAAAGAAGGCGAAGAAACCTTCAAAGATCTGTGTTT
CATGATGTTCATGAACACAGAGTTCAAGATCTAAGTGCGGGGACTAGATCG
AGAACGGTGAGAAAATCACGCTATTCGGGCGTAGATTAGCGC

>dsRNA2

TGATAAAAATAGAAGTTCAATTCTAATTTTTATCCTAGTGAAGTTGCCACGTTAAACAACTCT
GAGACAAAATGGACAAGTGGAATCCGAAGAAGTATCAAAAAGTGGAGGAGACTTTCCAGC
TGCTAAGGTCGAGATCTCGATATGTGCAGCAGTTGCAAAAGGATGAAAAAGAGAAGAGAG
TGTGGAATCCATCTCGCTATGACAATTCAAAGGATTACGCAGGAGGAAGAGGATCTATAGGC
CAGGTGATATCGCACATGCAAGACTACTTGTCAAAGTATAGTGTTGGCGCTATGGAAGTGCT
GTCTGAGGAGAACATAGATGTGTGGTATATGATAATGGGTGATCTTGAAAGAAAAGCGAATC
TGGGAGAAAACACATGTGGCATAAGAGCGGATTTCGAGTGGACACAGATGGCGTGCAGTGT
GGTGACGCACGCCGGGCAGAACACTGAATTGGCATTTAGTGCGACGACAGCTGAGGTGATA
AACAAAAACATGAATGACAGGGCGAAGATACAAAAAGCAACGGGATTTGAGATGCCAGAC
GTCCACAATATGAGGAGTGGAGATGCCGAAGATTTGAAGCGGTTGATACTTGCAGCTGCCG
GAGGTCAAAACAAATTCACGAGACTTGTGAAGGGTATGAGTCTGTATATAGATATGCTAATA
TATGGGAACCAAAGGTTTGAAGGCCAGATCAAGAACKCTGCCGAATACAATCTGCAGAACG
TTCTAGCTGCATTTAGGGCCAAGGACAGATCGCATTCATATTGCTCAACCGAACATTCTGACC
CGTACTTCGTGTTGATGTACTTGATAGGGCTGGAGTACCCTTTCAATGGGAACAACATGAAG
TGCAGAGGCGCCACGAGGATTCCAGCCGATGCTGATGACCATTGGGTAATGGTACGCGGGA
CTCCTAACAAATCGGAGTTAAAGACTGGTCTGACCGCTAATGCGGTGTGGGCGGGTATGGTG
AGCTACGCATCAGAAATGGAGGTGTCTGATCAGCTAAGCAGTGCACTCATTGTAGCGGCTAG
CCTATACCAGAATCGATACCTGAGGGAAGTGTCTTTACCCAAGGTTGGAAGTACAATTGACA
TAATAAGGCCGGCGTTCCATGAAGTTAGCACTGACGATGCARAGAAACCTTATCTTGACGCG
AGCGCAGTAGTAGCGTTGGGTAGGGTTCATCAGTTGAATTGTTTCTTAGTGGCAAAGGATCT
AATAGTGTCAGCGCAACATTCAAGTGAGAATGGGCTGAACTACGAGGAGGTGGTGATGCAG
TACTTGAAGCAGGATGAAACTGTTATGAGTAGGATGGCTGAATGGTCATCAGGATTATCGCY
GCWAGRSSCCACGAAGCAGATGAGGTGGATGGGGTGTCTTGACAGAGAAGATATACAGGAT
CTGAGTGCTATATCAATCTTTGAAGCACTATGGCTGTGTGATTCCACAACAAAGAGTGTAGA
GAGAGGAGCCATTGACTGTCTGGTGAGGGGAAGGAAAGACATGATGCCGAGGAACGGATA
TATTGAGCTGTTGGACGAAGAAATGCGCAAATTTGGAGTGCGACTTGACAGGTCCAGGATA
CCATCTGGGGCCTTCTCAGTGCAACGAAGGAGTATATGCTCAATGAACAGGTGGGCTCCTAA
GAAAAAGAAATGGGTTGAGAGTGATAGCAGCATGGTACAGCCTTGTGACTACGGTAAGTCA
GGTAAGAAGGAGGAGGTGAGAAGAAAAAGATTTTCTGTTAACCCACAGTCAGGCGACCAC
GTTTGGGAAGTGCACTACAAGGCCTATGCAGAAAGAAAAGCGAAGGAAAGGACACCTACA
CCTTCTAGTTCGGATGTGGATGAAAGTCCAAAAAGGAGGTCTTTTGATGGGAAAAAGATTGT
AGGGCTAGAGTTGCCACGCTTTCCTGGCCAGGTAGAGAGGAATGTGACTATGGTTGAGGTA
AGAAGTAGTAAATTGGGAGCTTCGGAGTACGGTGAAATAAGTGACGCTGGGTCATCAGAAG
TTGAGAGGATAGAGGCAGAGAAGCAAATGTTCGAGGAAGAGGAAGCACAACAGGAAGTA
GAATTGGTGCAGATAGACAACGATGGAGCATTCGGAGAAGTGTTGAGAAAGAAGGGCAGC
AAACAGAAGAAGAAAGTGAGTTACGCAGAATTCATGAAAGAAGAGAATCCAATTAATGAT
GAGCAGCAGGCAATACACGAAGGGAAGGAATACCACGGGGTAATAGATTGGTCATGGGAC
CCAGACACATTGTGGAGGATAAGGAACAGCAAGTCGGTAGGTGAGTTCTTAGATGATAAGG
GCCTAAGCAGAGGGAGCACGATTCTTGGGCTCTCAAGAGAAGAATTCTTCGGAGAGGCAGT
TAGGCACAGTCTGATAATGAAGCACCTAGATGTTACGATAGTGACATTTAAGGAGAGTATGA
CTAGAGTGCCTATGTATGCACTAGAAATGGCATTCGTGTCAGAAGTGGTATTCGGACTGAAC
GCCAGAGAAAGGCAGAATGAAGATAGCTTTGAAAAAGGCAAGCTGGGATTAGGTGACGCG
TATGTGGCAGACCTTGTACACAAGAACGATATGGTGGCCGAGTCATTGTCTAAAGTTCAGAG
AGAGAGGATCAATAGGCAATACAAGGCAACCAAGAGAGAGATAGCAAGGCTGAAGGAATT

TAGTAGGCGATCTGTCTCAGGCTCTGATAGTTCTAGGGGTATTGTACAGTACTCAATAGAATC
AGATGCAAGGTCAATCTAGCATGTTTATGCTAGATTGATATCCTAAGTGCGATGTGGGAAGG
CTCGCATGAGGGACGGTGAGAAAATCACGCTATAGGGGCGTAGATTAGCGCA

>dsRNA3

TGATAAAATTGGAAGTTATCAATCCGTTTTATCCTAGTGAAGTTGCCACGTAAAACAACTCTG
AAGATGGCCCAGTTCAAGACTGTGTATGATCTTATGGGCGATGCAGCGAAGGGGAAGTTCTT
TAGGGGTAAGAGCATGAGCGAAAGTGCAGTTGATGAGATGGAACATGTTGATTGTGAGTTT
GAAGAATTCAAGTCGTTGGGGTTGGTTGAGAAAAATATCCTTGCAGGAATAAGCATGCCTG
ACAATTTCACAGTTAAGAAGTCAGAGGGTAAGGTCGACATACTTGAGATTGGCATGACAAC
ACTGGCCAAGGCGGTGAGTGAGCACGGGGGTGTCACTGATCTAACGCACACAGCTGTCAA
GAAAATAGATCCTTTCCGAGTGTATGGAGACTGGAAATTGGAATATGAGCACGGCAGCATAA
TGTATCCTTGGCACAAAGGGATAAAGGCAGACAGGGACAAAGGAGTAATGGGCAGGATAT
CGGCACAATTCCCGCATTTGAACTCGACAGAGTCAAGCGGTTTTACAAGAACACTGCTCAC
ACATATGCTAGCGCAGAAAACGGACATGACACACGTGTTCATGAAGGGATATCTGATAATGA
TGGACTTGAACACGTCGGTTGTATTCAGACGCACCATAAGAAGAGCAACGGCAAAGAGAG
ACAACTTCTATCCAAACAAGAGTAATGATCATAGGATGGGACTGGTTGGCGCAGCTAGAATA
GTTGTGGACGCAGATGGATTCTCAGCTGAGGAGCTAGAATCTCTGGACATGATGTGCCAAG
AGTATCCGACAGTCAAGTACTGCGAAGATAACATATACAATACATGCAACATGGAGGCAGA
CAGGTTGGCCATAACAAGTAGCCGTGAGATACCTAGGGCACAGGGTAGAATTCCAACTCCA
CTGGAGTTTTACAGGTTGATGACCAGTATTGCATGTAAGCTGGACGCAGTGGAGGACATGTT
CACAGCCTTCAAATTGATGAGAGGACGGATGGCACACATAAGAGATGTTTACAGGTCATCA
GACGATAGGAAGTACTCCAGTGGCGTGCCACTTAGCACATGCTACTCAAGGTGCCTTGGAG
GAGGCACAGTGCACAAGAAAGTCTGTGCAACATACCCTGCTTACATGTGCTCTAGCGTGGG
ACTTGTCGCGGACGTACTGCTAGGTAGGTGCTATGAAGTCGCAGCGTCCATGCTTATAGAGA
ATTTGGGCGGACTGGGAGACATGATGTGCAGTGGAGGTGCGAGGGCAAGTGGACAATACA
ATGGGCTACTTAGAGAGTATGGCCTTTCCACAAAAGATGCAACAAAGAATGAGCTGATGTT
GGCGTGGGAAGGCATAGGCGCAATTGAGTACAAGTGGAGCCCAACTCTCACATGGAAGCC
ATACATAGTTAAGTTGACAGATGAAATAAGAGGAGGAAATGACGTAGAAATGCCACAACTG
TGCTATGAGATCGCGCACATGTTCCACGTAGACAACGCCTGGGGCGCAATACGGGGATATAA
AGGAATAACAGGAGCAGGTCTAGCAGAACACACCGACATAGGTGAAGGAGCAAGGAAGA
TGAAAGATGAAAGGCTACGGTATGCAGCAGCTTTCTCTTGGGCAATGGGAGTTAGGCGACA
GCGGCCCAAGATATTTGTAAATTCTTACGGAATGAAGGAAGTGGCAGTGTCAACTAATGAG
AGGAAATTTCTGAGGTCAGCAATAGGGGGGTACAAATTCACCAATTTGTCCTACACCCTGGA
AGAGGATTATGAAGGAAGAGAGGACTGGCTGGAGAACGCAGCAACTGAGCTGTTCAGGTC
AACTATAGATGGGACCAGGTGTTCGGTGATAATGTCAACGGCTGGAGAATGGCATTATTCAG
AGACCAAGGAGTCGAAGACTGAAGGAATCGCTGAGTCTCTGATGCAGAAACAAAAGTCTG
ACAATGTGGACGAGGAGATTCAGAGGATGACCACTGGCAAGTTAATAGAGCCAACAAGCA
ATGGAGCTGGAGATCTGTTCAGCAAGATGAGCAAGACAAGGTCTGTGGTCCACACACCCAA
AGTTAAGTTGTCAGGAGGATCGCTGCCAGAGGTGAGAGAGGGAGAGACGAGCTTCAAAAC
AATACCTGTTCCTGGTGATGGCAAGTGTGGTATACATGCAGTTGTTGCAAGCATGAAGGAGG
CAGGCATGCTTAGAATGGGTGAAGAGAAATTGGTATTCGACAATTTTGACGGCCAGT
TCAATGGGCAAACTTTCCACGATGCACAAACAATAGCACAGGGTCTGAACGAGATAGGGAT
CGGGCTTAGGCTGTATGATAATATACCAGAAAAGGGTGTGAGAGTAACGGA
CTATGGGGATGTGAAGGAGAATGGTATAGCAATATATAGGTCAGGCAACCAC
TTTGAGGGTGTAGTGGAGGGCCTGGGCGATACTAGAACAGTGATAGCTAAAGAA
GGAGGACAGCTGTCAAATGTTCAACAGGTAGACGCACTCAAAGAAATGAGGAG
ATTCTTTGAATAACACTGAAGCGTCTGCGGGGGTGACTCGACCTTGGTGTGT
ATCCTGAGGTGCGAGTTGCTGGAGAAAATTCTGAGTGGGGTCAGGGTTAGCGC
